# Exploring how residential care facilities can enhance the autonomy of people with dementia and improve informal care

**DOI:** 10.1177/14713012211030501

**Published:** 2021-07-02

**Authors:** Jogé Boumans, Leonieke C van Boekel, Marjolein EA Verbiest, Caroline A Baan, Katrien G Luijkx

**Affiliations:** Tranzo, Tilburg School of Social and Behavioral Sciences, 120694 Tilburg University, Tilburg, Noord-Brabant, the Netherlands; Tranzo, Tilburg School of Social and Behavioral Sciences, 120694 Tilburg University, Tilburg, Noord-Brabant, the Netherlands; Ministry of Health, Welfare and Sports, The Hague, the Netherlands; Tranzo, Tilburg School of Social and Behavioral Sciences, 120694 Tilburg University, Tilburg, Noord-Brabant, the Netherlands

**Keywords:** autonomy, informal care, dementia (care) person-centred care, realist evaluation, physical environment, interactions, technology

## Abstract

**Background and objectives:**

Residential care facilities (RCFs) strive to enhance autonomy for people with dementia and to enhance informal care provision, although this is difficult. This study explored how RCF staff can enhance autonomy and improve informal care by looking at the influence of interactions (contact and approachability between residents, staff members and informal caregivers) and the physical environment, including the use of technologies.

**Research design and methods:**

A realist evaluation multiple-case study was conducted using document analyses, eight semi-structured interviews with staff members and relatives and 56 hours of observations of residents across two RCFs aiming to provide person-centred care. Realist logic of analysis was performed, involving Context-Mechanism-Outcome configurations.

**Findings:**

The behaviour, attitudes and interactions of staff members with residents and informal caregivers appeared to contribute to the autonomy of people with dementia and enhance informal care provision. The physical environment of the RCFs and the use of technologies were less relevant to enhancing autonomy and informal care provision, although they can support staff members in providing person-centred care in daily practice.

**Discussion and implications:**

The findings add to those of other studies regarding the importance of interaction between residents, staff members and informal caregivers. The findings provide insight for other RCFs on how successfully to enhance autonomy for their residents and to improve informal care provision, as well as, more broadly, how to implement person-centred care.

## Background and objectives

Many traditional long-term residential care facilities (RCFs) for people with dementia have changed their focus in recent decades from physical care, risk reduction and safety (White-Chu et al., 2009) to acknowledging the importance of ensuring that their care provision aligns with the preferences of residents (de Boer et al., 2017; Kitwood, 1997; Mitchell & Agnelli, 2015). More RCFs are developing and innovating to shift to a more person-centred care approach. That many RCFs have their own strategies and ways of implementing person-centred care makes it interesting to explore which elements contribute to the diverse aspects of enhancing person-centred care.

Providing person-centred care involves ensuring that the people who are receiving care can make their own choices. However, facilitating the autonomy of people with dementia living in RCFs is rather complex (Bennett et al., 2015; Welford et al., 2012). Such people could face difficulties voicing their needs and wishes, and they may need support from others to express and execute their autonomy. RCF staff must therefore be aware of the fact that although people with dementia may not have the capacity to carry out a decision, they maintain the right to be involved in the decision-making itself (Kitwood, 1997; McCormack, 2001). To gain insight into how RCF staff behaviours enhance the autonomy of residents with dementia, we chose a definition of autonomy used in relation to person-centred care. This definition has two elements: (a) *decisional autonomy,* which refers to the ability and freedom to make choices, and (b) *executional autonomy*, which refers to the ability and freedom to carry out and implement those choices (Collopy, 1988; McCormack, 2001). Next to autonomy, encouraging relatives to provide informal care (unpaid care and support; see Reinhard et al., 2008) for their relatives living in an RCF is essential to providing person-centred care (Edvardsson et al., 2010; Natan, 2009). Relatives are familiar with the likes and dislikes of the people with dementia (Eurocarers, 2009; Reid & Chappell, 2017), and having them involved in care could lead to more person-centred care for the RCF resident with dementia. The current study focuses on these two essential elements of person-centred care: autonomy and informal care provision (Edvardsson et al., 2010; McCormack, 2001).

The involvement of people with dementia themselves and informal care providers in healthcare provision is important for developing and carrying out appropriate (care) plans. The formation of good relationships between staff members, residents and relatives is thus an essential aspect of maintaining autonomy and increasing informal care in RCFs (Boumans et al., 2018; McCormack, 2004; Natan, 2009). For the purpose of this study, we refer to contact and approachability in the triangle between staff members, residents with dementia and informal caregivers as ‘interactions’.

The physical environment of the RCFs is also important for stimulating residents’ autonomy and informal care provision. Unit size, spatial layout and homelike character improve resident autonomy and also influence the provision of informal care (Chaudhury et al., 2018; Day et al., 2000). An important element of the physical environment is the use of technology. For example, GPS trackers allow residents to move freely within and outside the facility and therefore contribute to their autonomy (Gordijn & Have, 2016; Robinson et al., 2009). Digital technology also makes it possible to involve informal caregivers in a different and greater way in the caregiving process for residents of RCFs (Boumans et al., 2018).

In sum, for people with dementia living in RCFs, different policies and/or staff behaviours are important in stimulating resident autonomy and improving informal care. The objective of our study was to provide insight into *how* interactions, the physical environment and the use of technology in RCFs contribute to maintaining the autonomy of residents with dementia and improving informal care provision.

## Research design and methods

A realist multiple-case study was carried out to describe two RCFs and clarify how their care affects autonomy and informal care. Case study research is recognized as being particularly useful when the focus is on seeking answers to ‘why’ and ‘how’ questions (Yin, 1994). This approach is compatible with realist evaluation, which is a theory-driven method for understanding how and why complex interventions (such as those which depend on active staff decisions) work or fail to when applied in complex settings (Pawson & Tilley, 1997). Case studies appear to be useful to study health care according to the principles of realist evaluation (Mukumbang et al., 2018; Williams et al., 2013), which seeks to unpack the relationships between context, mechanisms and outcomes – that is, how particular contexts trigger (or interfere with) mechanisms to generate the observed outcomes. The *context* includes elements such as the organizational context, participant features, staffing and geographical and historical context. *Mechanisms* are a combination of recourses offered by the (social) programme or intervention and human understanding and/or responses to that recourse. Mechanisms are not directly observable and include preferences, reasoning, norms or collective beliefs. *Outcomes* include changes to people and to their lives, but also include other kinds of alterations (e.g. in organizations, workers or governments; Westhorp et al., 2011). The relationship between the context and mechanisms leading to certain outcomes is the so-called Context-Mechanism-Outcome configuration (CMOC). In this study, the organizational context was the RCF in which people with dementia live (features of participants). The outcomes (changes for people) were maintaining autonomy and/or improving informal care provision. We explored how and why interactions, the physical environment and the use of technology (organizational context) were triggering mechanisms (responses of people) and, as such, influenced autonomy or informal care provision (outcome). RAMESES II reporting standards for realist evaluations were followed (Wong et al., 2016).

### Data collection

Two RCFs in the southern part of the Netherlands took part in this study. Both provide long-term care for people with dementia and take a person-centred approach to care, but are not run by religious organizations. These RCFs were included in the study because they have different origins. RCF A is a newly developed RCF, whereas RCF B has been established in 1970. It is interesting to compare a new organization that could implement a person-centred care approach from the start/beginning with an organization that had to redirect their traditional care approach to a more person-centred care approach. RCF A (2015) is a new, innovative RCF based on hospitality-style principles. RCF A seeks to provide service such that older adults (55 years and older) can age in a pleasant way together with their partner. At RCF A, joy of living, comfort and hospitality (e.g. offering quality food and luxurious rooms and restaurants) are central aspects. Providing care according to the wishes and needs of residents and relatives is valued above following rules and procedures where possible. RCF B has a longer history (since 1970), a more traditional design and has gradually shifted over time towards a person-centred care approach. RCF B states that everyone is able to participate: residents, family members and people from the community can join and participate in the care and support of residents in a way they prefer and are able to. Both RCF A and B qualify as small-scale living facilities within a larger nursing home (de Boer et al., 2018). They both provide care for people with dementia in a homelike situation. A maximum of eight residents form a joined household. A common living room is provided, which includes a kitchen in which all meals are prepared. Facilities such as a restaurant and activity areas are attached to the ward.

Several data collection methods were used consecutively to understand *how* the interactions, physical environment and use of technology in both RCFs contribute to residents’ autonomy and informal care provision. Using different data collection methods consecutively made it possible to incorporate the knowledge gained in earlier phases into subsequent phases. In the first phase of data collection (April–May 2017), relevant documents such as vision papers, annual reports, policies, reports, meeting minutes, newsletters and other documentation were collected from both RCFs (A, *n* = 13; B, *n* = 24) to provide data about the organizational context.

In the second phase (May–July 2017), non-participatory observations of residents with moderate to severe dementia, two from each site (*n* = 4), and staff, two from each site (*n* = 4), were held. The contact person of each RCF selected several staff members and legal representatives of people with dementia and informed them about the study with a letter, written by the researcher (JB). All participants who were willing to participate returned a signed informed consent form directly to the researcher and were contacted by phone and asked when the observation could take place. Residents were informed and asked for permission by the observer and a staff member about the observations prior to the observation moments. All residents were able to respond verbally and agreed to participate. We developed an observation guide based on our earlier review and the dimensions of observation (space, actor, activity, object, act, event, time, goal and feeling) suggested by Spradley (1980). The observations focused on how elements of the interactions, use of the physical environment and technology by people with dementia and informal caregivers contributed to the autonomy of people with dementia. Observations of the residents and staff took place both during the day (7 a.m.–3 p.m.) and evening periods (3 p.m.–9 p.m.), Monday to Saturday, and took on average 3.5 hours. Notes were taken while conducting observations, and these were typed up after each observation day using the observation guide.

In the third phase (November–December 2017), semi-structured interviews were held with various people: board members of the RCFs, two from each site (*n* = 4); staff, two from each site (*n* = 4); and informal caregivers/relatives of residents with dementia, two from each site (*n* = 4). The board members who were most knowledgeable about issues related to the subjects of the study (i.e. the design for caregiving at the RCF and which elements could enhance autonomy and information care provision for residents with dementia) were invited to participate. The contact person of each RCF selected several staff members and legal representatives of people with dementia and informed them about the study with a letter, written by the researcher (JB). Participants who were willing to participate returned the informed consent form directly to the researchers and were called and asked when an interview could take place. Staff had knowledge about the context and for whom the way of caregiving works. Informal caregivers/relatives know how and why the care provider the care provided is working for the resident with dementia (Manzano, 2016). The aim of the interviews was to unravel how the RCF as an organization – and in daily practice – maintained the autonomy of residents and improved informal care provision. Additionally, noticeable and unclear findings from the document analyses and observations were discussed during the interviews.

In the fourth phase (December 2017), a second round of non-participatory observations took place with staff, two from each site (*n* = 4). The same observation method as in the second phase was used, although more focus was given to the specific details of the performance of regular care tasks, such as helping residents shower and dress. This strategy was chosen to unravel subtle differences between the caregiving approaches of both RCFs in these specific situations. Observations took place during the early morning (7 a.m.–11 a.m.) or late evening (9 p.m.–11.30 p.m.).

### Data analyses

The interviews were transcribed verbatim, anonymized and coded using Atlas Ti (version number 7.5.18). Documents, observation reports and transcripts were coded by two authors (JB and LvB) independently concerning how interactions, the physical environment and use of technology contributed to the autonomy of people with dementia and to the improvement of informal care provision. The results were discussed until a consensus was reached. For both RCFs, the interactions, physical environment and use of technology to support autonomy for people with dementia or informal care provision mentioned in the documents, observations reports (both phase 2 and 4) or interviews were summarized in an Excel file. Based on this summary, CMOCs were drafted.

### Ethical considerations

Research ethics committee approval was granted by the Tilburg University Ethics Review Board (ERB, ref EC-2016.68). We also obtained ethical clearance from both research sites.

## Findings

Table 1 provides an overview of the general characteristics of both RCFs. Although RCF A and B provide 24-hour care for people with dementia, RCF B provides care primarily for people with moderate to severe dementia, whereas RCF A also provides care for people with light to moderate dementia or somatic problems. People are eligible to reside and receive care in RCF B if they have a diagnosis of dementia and are in need of long-term care. To live in RCF A, an indication for long-term care is needed, but a diagnosis of dementia is not necessary. The findings below are presented as CMOCs, which we have themed by elements of the RCF – namely, interactions, physical environment and use of technology and the influence of these elements on outcomes, autonomy and informal care provision. The CMOCs are explained and illustrated by concrete findings from the data collection in the RCFs.

**Table 1.** Characteristics of the two study sites.

	RCF A	RCF B
General characteristics		
Founded in	2015	1970
Location	City in urban area in the southern part of the Netherlands	City in urban area in the southern part of the Netherlands
Type of residential care facility	No closed care ward (no registration under the Dutch Exceptional Medical Expenses Act)	Closed care wards
Type of care	Long-term care/rehabilitation	Long-term care
Physical environment		
Building	Two floors within a six-storey building built in 2015	Three-storey building built in 2013
Number of apartments	85 care apartments 200 regular apartments	54 care apartments
Reception	Hotel-like bar	No reception
Outside area	Garden with goats and chickens	Courtyard
Facilities	Three restaurants and a café, hairdresser, grocery store, swimming pool and children day care	A grand café, hairdresser and ‘snoezelen’ bathroom
Design of care unit	Small-scale,^a^ homelike care facilities, two or three living rooms next to each other connected via an internal door, residents’ apartments located in long hotel-like hallways	Small-scale,^a^ homelike care facilities, two living rooms on each floor, residents’ apartments located around the living rooms
Design of living room	Seats for eight to 10 residents, open floor plan kitchen, dining table, seating area with television and a balcony	Seats for six residents, open floor plan kitchen, dining table, seating area with television and an indoor balcony
Design of residents apartments	On average 44 m^2^, kitchenette without cooking area, a living/sleeping room and a private bathroom with toilet. Some apartments have a balcony.	23 m^2^–55 m^2^ apartments, a living and sleeping room; en suite bathroom with toilet is shared with the neighbouring apartment.
Staff characteristics		
Number of staff in full time equivalent in 2017	74	59
Care staff	24 hours a day: nursing assistants Daytime: licensed practical nurse, nursing assistants and interns	24 hours a day: nursing assistants Daytime: registered nurse, licensed practical nurse, nursing assistants and interns
Welfare staff	Welfare guardians do not perform care tasks and do not necessarily have education in health care. They assist during the daily lives of the residents in and around the living rooms (eating, drinking and activities). From 8 a.m. until 10.30 p.m., there is one welfare guardian in every living room. Between 2 and 4 p.m., two welfare guardians are present to perform extra tasks, such as walking outside with the residents. Buddies*:* resident gets assigned two staff members who function as ‘buddies’. Buddies are the first contact person for residents and relatives. Their task is to get to know the residents’ preferences and to inform the other staff about them.	Hostess works in two group homes between 8:30 a.m.–12:30 p.m. and 3:45 p.m.–7 p.m. They do not necessarily have education in health care and do not perform care tasks, but assist the residents with breakfast, lunch and dinner and perform domestic chores.
Resident characteristics		
Number of residents	56	54
Number of residents with moderate to severe dementia	*n =* 25 (45%)	*n =* 48 (89%)
Profiles of residents with moderate to severe dementia	Extensive need for 24-hour care	Extensive need for 24-hour care
Technology use		
Technologies	Eclectic trapeze bar (against payment) in the room Care watches (against payment) equipped with an alarm button Tablet device in apartment with alarm	—
Records	Tablet to review and update residents’ records	Tablet to review and update residents’ records
Freedom of movement	Transmitter in watch (against payment and should be approved by relatives)	Tag on clothes (should be approved by the relatives)

Note: RCF: residential care facility.

^a^Small-scale living is provision of care organized around small groups (approximately eight residents); residents and staff form a household together, so daily activities (cooking, cleaning, etc.) are integrated within daily care (de Boer et al., 2017).

### Interactions and autonomy

We identified five mechanisms for interactions that enhance the autonomy of people with dementia. Two of them contribute *directly* to autonomy: enabling residents to make their own choices and enabling residents to carry out own choices. We also found three mechanisms that might indirectly lead to more autonomy for residents: social inclusion, staff’s respectful approach and knowledge of residents.

#### CMOC (A1): enabling residents to make their own choices

Both RCFs have policies aimed at enabling residents to make their own choices, such as asking residents their opinion *(context).* This form of decisional autonomy was observed in both RCFs, where staff consulted people with dementia before they acted, and staff considered the opinions of the residents seriously in most situations *(outcome),* which is illustrated by the following observation:

It is 5.30 PM and most of the residents are sitting at the dining table. The staff member is preparing a meal. According to the menu, boiled potatoes should be served, but Mrs X has indicated that she prefers French fries. Therefore, the staff member orders French fries from the restaurant in the building. (Observation RCF B)

In the interviews, staff as well as relatives at both RCFs indicated that staff enable residents to perform activities and the freedom to make their own choices *(outcome).*

##### Explanation of the mechanism

RCF policies to enable residents to make their own choices (*context)* could result in staff consulting residents before they act and also in taking the opinions of residents seriously *(mechanism)*, which confirms that the decisional autonomy of residents is being respected *(outcome).*

#### CMOC (A2): enabling residents to carry out their own choices

In the documents from both RCFs, policies were found regarding residents making decisions and acting upon their own choices *(context).* We observed that residents in both RCFs were better able to perform activities themselves *(outcome).* However, because of the hospitality-style approach *(context)*, the staff in RCF A apply a restaurant-style approach to serving food and drinks. Coffee and tea were served, including milk and sugar. Lunch and dinner were prepared by staff and served on plates, comparable to a restaurant. Residents thus had less opportunity to execute such tasks themselves *(outcome).* The hostess and staff in RCF B also make coffee or tea for residents, but place milk and sugar near the residents so that the residents have to add it themselves. In cases where residents were able to serve coffee themselves, they were encouraged to do so. Lunch and dinner were presented plated or in bowls, and residents served themselves *(outcome).*

##### Explanation of the mechanism

If RCFs encourage residents’ executorial autonomy though policies *(context)*, staff members may be more likely to encourage residents to perform activities themselves or to help residents implement their choices *(mechanism).* This leads to residents experiencing more executorial autonomy *(outcome).*

##### Explanation of the *counter-*mechanism

RCF A demonstrates a different outlook on executorial autonomy regarding eating and drinking. The view of staff working in an RCF based on the hospitality-style approach *(context)* could lead to staff members carrying out eating and drinking support activities for residents because staff members find it important to be hospitable *(mechanism)*, which yields less executorial autonomy for residents regarding food and drink activities *(outcome)*.

#### CMOC (A3): social inclusion of residents

Document analyses of both RCFs revealed policies for social inclusion of residents (residents are given the feeling they are full members of the social group): (a) staff do not wear uniforms; (b) staff join residents at table for meals; and (c) staff have to include residents in activities (not only activities especially designed for them but also in household chores like peeling potatoes) *(context).* During our observations in both RCFs, we established that no staff members wore uniforms and the term ‘nurse’ was seldom used. Staff included residents in tasks, such as setting the table, making dinner and folding laundry. During meal times, staff sat with residents and included them in conversations about everyday things such as the weather or events in the city. Residents enjoyed having a meal together under these circumstances. We also observed that staff included socially residents by speaking directly with them and treating them as people instead of patients *(outcome)*. During conversations with the residents, the staff members’ tone of the voice was not patronizing but respectful and the topics were relevant for the residents, such as (previous) holidays and having children.

##### Explanation of the mechanism

RCF policies regarding the social inclusion of residents, staff not wearing uniforms and staff and residents performing activities together and dining together *(context)*, as well as the tone of voice used by staff and the topics discussed between residents and staff, could lead to staff members looking and acting as though in an ordinary social situation. This could lead residents to feel like a member of a social group *(mechanism).* This staff approach may contribute to greater person-centred care *(outcome),* which in turn could contribute to encouraging resident autonomy.

#### CMOC (A4): respectful approach of staff

The analyses of documents from RCF A and B revealed that both RCFs consider the way staff approach residents to be of great importance. RCF A focuses on a hospitality-style, respectful approach that should facilitate residents in living the life they desire. RCF B emphasizes a ‘welcome, homelike and a respectful’ approach regarding resident preferences *(context).* The observations confirmed this approach; staff asked for approval from residents before acting, knocked on doors before entering and considered resident preferences. In addition, residents’ questions were answered in a respectful manner, even when a resident asked the same question multiple times *(outcome).* During the interviews, the relatives of residents at both RCFs expressed that they were pleased with the approach of the staff because the tone of interactions between the resident and staff is social and respectful *(outcome)*.

##### Explanation of the mechanism

RCFs have policies regarding the staff’s respectful approach to residents to facilitate residents in living the life they desire *(context).* Resident preferences about their lives are considered as much as possible by the staff *(mechanism).* This approach may contribute to greater person-centred care *(outcome)*, which could in turn enhance resident autonomy.

#### CMOC (A5): knowledge of the resident

Analyses of documents from both RCFs revealed several policies focusing on the importance of staff members getting to know the preferences of residents by being involved in the residents’ lives *(context)*. Observations in both RCFs showed that the staff members who work with residents on a regular basis are familiar with residents’ needs and preferences *(outcome)*. Furthermore, during interviews, relatives acknowledged that staff at both RCFs possessed a lot of knowledge about the residents *(outcome).*

##### Explanation of the mechanism

RCF policies to encourage knowledge of the residents *(context)* could lead to staff members showing motivation to get to know the residents’ preferences and to act upon this knowledge *(mechanism).* For residents, this could mean that care delivery is personalized to their needs and preferences *(outcome)*, and this person-centred care delivery may facilitate residents’ autonomous choices.

### Interactions and informal care

The importance of informal care is acknowledged in the policies of both RCFs. Staff members at both RCFs enhance informal care provision, which contributes both *directly* and *indirectly* to more informal care provision.

#### CMOC (B1): encouraging informal care provision

Both RCFs have policies actively to involve informal caregivers. In our document analyses, we found that RCF B explicitly states the types of care tasks in which staff can involve relatives for informal caregiving, such as taking a walk with residents. RCF A emphasizes the importance of a fixed contact person for relatives. This contact person is a member of the staff and actively involves relatives in informal care tasks by explaining what is expected of them *(context).* During our observations, we saw some instances in which relatives provided informal care. In RCF B, relatives helped with residents’ laundry or prepared a sandwich for them. In both RCFs, relatives took residents on a day out or went on a holiday with them *(outcome).*

##### Explanation of the mechanism

RCF policies to enhance informal care provision could lead to staff members explaining why the help of the relative is needed *(context).* If relatives understand why their help is needed *(mechanism)*, they may be more willing to perform such informal care tasks *(outcome).*

#### CMOC (B2): positive attitude of staff regarding informal caregivers

Both RCFs use different methods to encourage staff to have a positive attitude regarding informal caregivers. Both RCFs have policies regarding a welcoming (RCF B) of hospitality-style attitude (RCF A) towards informal caregivers. RCF B offers staff a course on the importance of informal caregivers *(context)*. We observed that, although relatives were welcomed by staff members in both RCFs, relatives in RCF A were offered coffee by staff, whereas in RCF B staff encouraged relatives to make coffee for themselves and their resident relative to create a homelike environment *(outcome)*.

During the interviews, relatives of residents at both RCFs gave positive feedback about the attitude of the staff towards them: ‘*I have many acquaintances who visit my mother when I am not there (…). I tell them to ask the welfare guardian for a cup of coffee (…). And the welfare guardian brings two cups of coffee with biscuits to my mother’s room (…). The atmosphere is really very warm and welcome (…)’.* (Interview relative RCF A)

##### Explanation of the mechanism

If RCFs encourage staff to have a positive attitude regarding informal caregivers *(context)*, this could lead to staff showing a positive attitude towards relatives. When relatives feel welcomed in the RCFs *(mechanism)*, they feel free to perform activities for the relatives *(outcome)*, which may result in (an increase in) informal care activities.

### Physical environment and autonomy

Although RCF A offered a wider range of technology use, most was only available at a charge and most residents of RCF A thus did not use this technology. We therefore could not observe the use of this technology, and the difference between RCF A and B with respect to used technology was very small. We identified two mechanisms related to the physical environment that directly contributed to enhancing resident autonomy: design of the physical environment and private rooms.

#### CMOC (C1): physical environment design enables freedom of movement

Both RCFs have policies involving freedom to walk around on the premises. In both RCFs, most doors are open. Nevertheless, the front door of RCF B is locked for residents because RCF B is a closed ward. In RCF A, the front door was not locked because it is not a closed care ward, although staff members were alerted when residents walked out the front door and guided the residents back to the ward *(context).* During our observations, we did not see any signs of agitation regarding freedom of movement among residents at either RCF. Residents of RCF A have the option of wearing a GPS watch that detects their movements. We observed that the physical environment enhanced resident autonomy inside the ward because it allowed residents to move around freely there. For example, a resident in RCF A was able to visit a fellow resident who sat in another living room, and they had a cup of coffee together. A resident in RCF B was able to take the elevator downstairs and use a scale because she wanted to know her weight. In the interviews, none of the family members reported problems regarding restrictions to freedom of movement. During interviews with staff working in RCF B, they reported that greater freedom of movement requires good communication amongst staff regarding the location of residents. A staff member of RCF A explained that they are trained to approach residents in a respectful and friendly way when they walk outside the care unit to guide the resident back to the unit. There are the differences between RCF A (open ward) and B (front door locked) on paper in terms of freedom of movement, but due to the actions of RCF A staff members, no differences in autonomy were found, and there were no great differences in where and when residents chose to move inside the wards (outcome).

##### Explanation of the mechanism

In RCFs that provide residents freedom of movement through a combination of open doors, monitoring options and good communication between residents and staff *(context)*, residents are able to go where they want within the RCF *(mechanism)*, which facilitates residents’ autonomy to choose where and when they want to move *(outcome).*

#### CMOC (C2): private rooms

Both RCFs have policies about respecting resident privacy. In both RCFs, residents have a special key or key card to enter their private room. Staff members at both RCFs are asked to respect the privacy of residents by knocking on the door or ringing the doorbell and waiting for confirmation before entering *(context)*. During observations, we saw that residents in both RCFs spent some time in their own room *(outcome)*. In RCF B, a resident only joined other residents for meals in the shared living room upon invitation of the staff. She preferred to stay in her own room and do crossword puzzles. In RCF A, we observed a resident who spent the afternoons in the shared living room and the evenings in her own room embroidering pillows*.*

##### Explanation of the mechanism

RCF policies about respecting resident privacy lead to residents having their own key for their private rooms and staff having to knock or ring the doorbell before they enter the room: no other person can enter the private room *(context).* This could lead towards residents experiencing privacy (*mechanism)* and facilitating residents’ autonomous choices *(outcome).*

### Physical environment and informal care

We identified one mechanism related to the physical environment that directly contributes to informal care provision.

#### CMOC (D1): providing access for relatives

Both RCFs created easy entry for relatives of the residents by providing them a key to enter the care unit (RCF A was closed from the outside to prevent unwanted visitors) and the apartment of their relatives *(context)*. Our observations showed that relatives regularly visited residents and sometimes also brought clean laundry or food. This indicated that granting access to relatives encourages relatives (as informal caregivers) to perform care tasks *(outcome)*, although this could be seen as undermining the privacy that residents were afforded. The RCF tries, however, to replicate the resident’s home situation, in which the family member often also has the key to the resident’s home.

##### Explanation of the mechanism

RCFs providing access for relatives to the care unit and apartment of the residents *(context)* leads to relatives being able to visit the residents easily *(mechanism)*, which could lead towards engaging relatives as informal caregivers in care tasks, such as doing laundry or bringing food *(outcome).*

### Use of technology and autonomy

Within both RCFs, we identified one mechanism where the use of technology could *indirectly* lead to more autonomy for residents by improving knowledge of the resident.

#### CMOC (E1): improving knowledge of the resident

In the RCF documents, the use of digital devices and digital dossiers was mentioned (*context).* While this study took place, we observed the use of tablets by staff to review and update residents’ records and care plans in both RCFs. In interviews, formal caregivers at RCF A stated that the use of tablets makes personal information of the resident easily accessible, also for (new) staff *(outcome).*

##### Explanation of the mechanism

The use of tablets in RCFs *(context)* enables staff to access the (personal) information of the residents *(mechanism)* easily, which enables staff to provide care that is personalized to the residents’ needs and preferences *(outcome)*. This may facilitate residents’ autonomous choices.

### Use of technology and informal care

Within both RCFs, we identified one mechanism which explained how the use of technology could *directly* lead to more informal care provision.

#### CMOC (F1): enhancing contact between caregiver and relatives

Staff in both RCFs used a computer program to report on the status of the residents. Relatives of residents at RCF A could also respond or ask questions using this system *(context).* Some staff members used the system to ask relatives whether they could help out with an activity *(outcome).* One relative stated during an interview: *[showing the computer program to the interviewer] ‘Look, here is a message from the caregiving team of my father: We are making plans for Christmas. You are invited to join us for coffee on Christmas Day at 11 AM. You can sign up (…)’.* (Interview relative RCF A)

##### Explanation of the mechanism

The use of a two-way communication computer system for staff and relatives *(context)* facilitates communication between them, which may enhance the ability of staff to involve relatives with care tasks *(mechanism)*, possibly resulting in more informal care provision *(outcome).*

## Discussion and implications

We performed a multiple-case study to explore *how* interactions between residents, staff members and informal caregivers; the physical environment; and the use of technology within RCFs contribute to resident autonomy and improve informal care provision. Interactions between residents, staff members and informal caregivers appeared to be most important in both enhancing the decisional and executorial autonomy of residents with dementia and improving informal care provision. An example of the effect of interaction between staff and residents on the executorial autonomy of residents was found in the way food and drinks were served. RCF A deliberately choose ‘service’ above autonomy for their residents, which means coffee and tea were served to them and dinner was served in restaurant style. In RCF B, however, residents participate as much as possible; residents were, when possible, supported in preparing their own coffee or tea, and food was served with plates and bowls as normal at home. Residents in RCF B were encouraged to serve themselves or at least to try to eat and drink by themselves. This may promote resident independence, which in turn may promote their decisional and executorial autonomy, despite cognitive or physical impairments.

The importance of the triangle between residents, staff members and informal caregivers in the care of older adults has been widely acknowledged (de Rooij et al., 2012; Mackie et al., 2019; Stanbridge et al., 2013). When staff members approach residents in a respectful manner, have knowledge of the residents and encourage autonomous behaviour, this leads to more autonomy for residents. This holistic approach of seeing the person instead of a patient is also recognized in other areas of care. Issel (2019) has noted that healthcare organizations that support staff members in seeing the patient as a whole person will likely have better patient outcomes. We also found that using this holistic approach of seeing the whole person, including context and history, also applies to enhancing informal care provision. Staff members who acknowledge the importance of informal caregiving for residents and who actively encourage relatives to perform informal care could enhance informal care provision. This finding is in line with another study (Wittenberg et al., 2018) which found that staff acknowledging the role and expertise of informal caregivers is essential to facilitate good collaboration.

Our findings indicate that, although less apparent, the physical environment and technology may facilitate interactions between residents and formal and informal caregivers and consequently contribute to the decisional autonomy of residents and to informal care provision. Using an electronic device to register medical data, as well as personal information about residents, makes it easier for formal caregivers to provide care that is in line with the preferences and wishes of residents, which may contribute to the decisional autonomy of people with dementia. Easy entry to the building for relatives appeared to enhance visits, which in turn may lead to more informal care provision. Other studies also found that technology may strengthen the knowledge of people with dementia (Thomassen & Farshchian, 2016; Webster & Hanson, 2014). Improving the use of communication technology between staff members and relatives appeared to increase relatives’ involvement and collaboration. Other studies have also found that technology can be used to educate and communicate with the relatives of residents living in an RCF to improve both quality of care and quality of life (Magnusson et al., 2005; Rosen et al., 2003).

Our findings are in line with the research of Santana et al. (2018), who has developed a framework for practical guidance on the implementation of person-centred care. This roadmap consists of three steps. The first step takes place at the structure/organizational level and relates to the context in which care is delivered. On this level, a culture of person-centred care should be created by developing and co-designing policies and education programmes and supporting staff to commit to person-centred care. The second step takes place on the process level and includes domains associated with the interaction between patients and healthcare providers. The third step takes place on the outcomes level and is reached when the value of implementing the person-centred care model in daily practice can be shown. When we apply the roadmap to our findings, we determined that when RCFs strive to create a person-centred culture by including certain expectations about the actions of staff members in (policy) documents (step 1), this may result in certain interactions between residents, staff members and informal caregivers in daily practice (step 2), which in turn may enhance person-centred outcomes (step 3), such as greater autonomy among residents with dementia and more informal care provision.

### Limitations and strengths

This study has some limitations. The severity of dementia experienced by the residents varied between the RCFs. In RCF B, almost all residents suffered from moderate to severe dementia, whereas in RCF A, several residents had mild to moderate dementia or no cognitive problems. For the purpose of this research, we only considered the residents with moderate to severe dementia. However, the difference in dementia severity was noticeable during our observations. The atmosphere at RCF A was livelier, and staff and residents with only somatic problems had conversations that were more ordinary and such residents helped with domestic tasks or even helped other residents with more severe problems.

The data collection took place in only two Dutch RCFs, and we observed a rather small number of residents and RCFs. Generalization of the findings may thus be limited. RCF A was founded in 2015 and was therefore relatively new when we conducted our study in 2017. The period between the start-up and data collection in RCF A was therefore short, and as a result, few policy documents, for example, were available. We would have preferred to include the opinions of residents with dementia themselves. Because of the focus of the study: how people with dementia living in an RCF are stimulated by the policies of RCFs and behaviours of staff to make autonomous choices or to perform autonomous actions, it was important to observe the interactions between residents and staff members and study if staff members were providing care according to the policies of the RCFs and, as such, stimulated residents to make autonomous choices or to perform autonomous actions. Observations were also a good research method because it allowed us to see the reactions of residents, including visible emotions*.* Involving staff members in the recruitment of participants may have introduced selection bias. Although the residents and staff were asked to maintain their usual routine, potential participant bias might have affected the outcome because participants may have behaved differently knowing that they were being observed. This could have led to more autonomy-supporting observations in which residents were better engaged in making or executing choices. Unfortunately, due to the methods used, it was not possible to assess whether or not residents themselves experienced more or less support for their autonomy during the observations.

Despite these limitations, this realist evaluation explored theories developed from the existing evidence and based them in case study sites with different cultures for dementia care. More or less the same mechanisms were found to operate at both study sites, which provided insight in the different aspects of person-centred care in daily practice. This could increase the transferability and usefulness of the findings beyond the local context, which could be used for other RCFs to implement a person-centred care approach successfully, with an emphasis on maintaining autonomy for their residents and improving informal care provision.

### Conclusion

By investigating the culture and policies as well as observing the daily practice of two different RCFs, we provided insight into how RCFs can successfully implement person-centred care to enhance the autonomy of people with dementia living in RCFs and improve informal care provision. The behaviour, attitude and interaction of staff members with residents and informal caregivers appeared to encourage the autonomy of people with dementia living in RCFS and enhance informal care provision. The physical environment of the RCFs and the use of technologies were less relevant in stimulating resident autonomy and informal care provision, although they could support formal caregivers to provide person-centred care in daily practice.
